# Effects of Environmental and Non-Environmental Factors on Dynamic Photosynthetic Carbon Assimilation in Leaves under Changing Light

**DOI:** 10.3390/plants12102015

**Published:** 2023-05-18

**Authors:** Yu-Ting Li, Hui-Yuan Gao, Zi-Shan Zhang

**Affiliations:** 1 College of Agronomy, Shandong Agricultural University, Tai’an 271018, China; 2 State Key Laboratory of Crop Biology, College of Life Sciences, Shandong Agricultural University, Tai’an 271018, China

**Keywords:** photosynthetic carbon assimilation, changing light, fluctuating light, higher plant

## Abstract

Major research on photosynthesis has been carried out under steady light. However, in the natural environment, steady light is rare, and light intensity is always changing. Changing light affects (usually reduces) photosynthetic carbon assimilation and causes decreases in biomass and yield. Ecologists first observed the importance of changing light for plant growth in the understory; other researchers noticed that changing light in the crop canopy also seriously affects yield. Here, we review the effects of environmental and non-environmental factors on dynamic photosynthetic carbon assimilation under changing light in higher plants. In general, dynamic photosynthesis is more sensitive to environmental and non-environmental factors than steady photosynthesis, and dynamic photosynthesis is more diverse than steady photosynthesis. Finally, we discuss the challenges of photosynthetic research under changing light.

## 1. Introduction

Light is the unique driving force of photosynthesis in plants; the rate of photosynthetic carbon assimilation is controlled by light intensity. Compared with other environmental factors controlling photosynthesis, such as temperature and carbon dioxide concentration, light intensity is distinguished by its more frequent, faster and larger variations. With the rotation and revolution of the earth, sunlight intensity changes daily and seasonally. In addition, the light intensity received by leaves also changes due to accidental events, such as wind, the shade caused by clouds and neighboring leaves and plants, etc. [1,2]. In terrestrial ecosystems, these changes in light intensity generally occur at temporal scales ranging from less than a second to several minutes.

**Figure 1:**
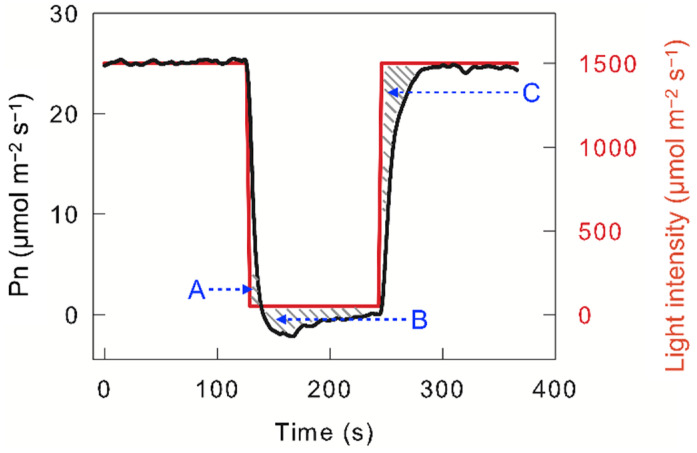
Light intensity and dynamic net photosynthetic carbon assimilation rate (Pn) in leaves of C3 species *Triticum aestivum*. The A area indicates post-irradiance carbon assimilation; the B area indicates the post-irradiance carbon burst; the C area indicates the loss in photosynthetic carbon assimilation in the photosynthetic induction stage.

Photosynthetic carbon assimilation changes with changing light intensity, which is known as “dynamic photosynthetic carbon assimilation” or “dynamic photosynthesis”. Dynamic photosynthesis does not fully conform to the change trend predicted by the light intensity response curve of the net photosynthetic carbon assimilation rate (Pn) [3,4], which is mainly due to photosynthetic carbon assimilation lag in response to light intensity. When high-light-adapted leaves were exposed to darkness or low light, photosynthetic carbon assimilation did not cease immediately but continued for a short time (seconds to tens of seconds); this excess photosynthetic carbon assimilation is termed “post-irradiance carbon assimilation” (Area A in Figure 1). Next, Pn decreased to a lower value than that observed under steady low light or darkness, which is due to carbon dioxide release during photorespiration; this excess carbon dioxide release is defined as “post-irradiance carbon burst“ (Area B in Figure 1). When the light intensity changes from darkness or low light to high light, Pn does not immediately reach steady high-light levels, but gradually increases in tens of seconds to minutes. This loss in photosynthetic carbon assimilation in the photosynthetic induction stage is reflected in the C area in Figure 1. Usually, the loss in photosynthetic carbon assimilation in the photosynthetic induction stage is much more than the post-irradiance carbon assimilation; hence, changing light usually leads to a loss in photosynthetic carbon assimilation. Combining the measured results with the prediction model, the loss in photosynthetic carbon assimilation caused by changing light may be as high as 20% or more [3,5]. Such losses unavoidably decrease crop yield. On the other hand, if the photosynthetic loss caused by changing light can be reduced, the crop yield is significantly improved.

The purpose of this review is to summarize the effects of environmental and non-environmental factors on dynamic photosynthesis under changing light. This review is aimed at beginners rather than experts. We hope readers can quickly understand the main fields and conclusions of dynamic photosynthesis research, and then determine directions for further literature reading and research. In the second section, we focus on seven common environmental factors, including the growth light environment, drought, salt, air temperature, air humidity, carbon dioxide concentration, nitrogen nutrition and circadian rhythms. In the third section, we focus on the influence of photosynthetic type (C3 and C4), inter-specific variations, intra-specific variations, stomatal behavior and specific genes on the dynamic photosynthesis under changing light. We only provide a brief explanation of the mechanisms behind these phenomena. Readers are encouraged to refer to previous reviews to understand the detailed mechanisms [6,7,8,9,10,11]. In the fourth section, we discuss the challenges of photosynthetic research under changing light. Here, we focus on photosynthetic carbon assimilation under changing light on a seconds-to-minutes temporal scale in higher plants. Research studies focused on higher light change frequencies [12,13], research addressing the effects of changing light on two photosystems [14,15] and research performed on algae were excluded.

## 2. Effects of Environmental Factors on Dynamic Photosynthesis under Changing Light

### 2.1. Growth Light Environment

Tang et al. reported that the leaves of *Quercus serrata* seedlings grown in a microsite with a lower sunfleck light intensity and a shorter sunfleck duration showed a more rapid photosynthetic induction [16]. Tinoco-Ojanguren and Pearcy compared the photosynthetic induction of *Piper auritum*, a pioneer tree species, grown on an open bench with (1% full sun) or without (60% full sun) a neutral shade cloth enclosure; the results showed that the photosynthetic induction was faster in shaded plants than in sunned plants [17]. Küppers et al. reported that the leaves from plants grown in more open regions took less time to become half-induced under saturating light than the leaves from plants grown in more shaded regions [18]. *Pseudotsuga menziesii* seedlings grown in the open had a higher photosynthetic carbon assimilation rate under steady light but slower photosynthetic induction than seedlings grown in the forest understory [19]. Nilsen et al. observed that photosynthesis reached 50% induction in 159 s for *Quercus rubra* seedlings grown in forest patches without shrubs compared with 226 s for seedlings grown in forest patches with shrubs present [20]. Durand et al. found that shade leaves of *Fagus sylvatica* complete full induction in a shorter time than sun leaves, but that sun leaves respond faster to high light than shade leaves due to their much larger amplitude of induction [21]. Wu et al. reported that increased planting density of *Zea mays* enhanced the change frequency of light and reduced the duration of daily high light; in addition, photosynthetic induction was significantly accelerated in *Zea mays* plants grown at a higher planting density [22]. 

In general, the above results show that plants or leaves grown in shaded regions have faster photosynthetic induction and maintain a higher induction state over longer periods in low light or darkness than plants grown in open regions. However, many different results have also been reported. Tausz et al. reported that canopy leaves had higher photosynthetic capacity under steady light, faster photosynthetic induction and took less time to reach 90% of the maximum Pn compared with coppice leaves of *Nothofagus cunninghamii* [23]. Leakey et al. reported that rates of photosynthetic induction were similar in *Shorea leprosula* and *Hopea nervosa* plants grown under long and short sunfleck conditions [24]. Sims and Pearcy showed that the understory species *Alocasia macrorrhiza* grown under short lightflecks, long lightflecks and uniform diffuse-light regimes showed similar steady photosynthetic capacity and lightfleck-use efficiency [25]. Research from Rijkers et al. showed that growth regimes (bright gaps and shaded understory) affected the maximum net photosynthesis rate rather than the photosynthetic induction rate in three shade-tolerant tree species [26]. 

In most experiments under natural or semi-natural conditions, the variation in mean light intensity and the change frequency of growth light were not completely distinguished. For example, compared with open sites, an understory environment usually represents lower mean light intensity and higher frequency change in light intensity [18,19,24]. Although in situ experiments provide abundant and real information, experiments performed in controlled environments are necessary. Qiao et al. reported that in *Zea mays* seedlings, a faster change frequency of growth light accelerated photosynthetic induction under high-light conditions following 3 min of weak light [27]. Tang et al. reported that plants grown under low light or lightfleck had faster photosynthetic induction than plants grown under high light [28]. 

It was also reported that photosynthetic induction was slower in *Arabidopsis thaliana* grown in sinusoidal high light than plants grown in changing light regimes [29]. However, Vialet-Chabrand et al. reported that plants grown under changing light conditions utilized the changing light less, not more efficiently, than plants grown under steady light [4]. It was also reported that the change frequency [30] and intensity of growth light [4] rarely affected dynamic photosynthetic properties in *Solanum lycopersicum* and *Arabidopsis thaliana*, respectively.

The above results indicate that the effect of growth light environment on photosynthetic response to changing light is species-dependent [31,32,33].

### 2.2. Drought and Salt

Drought stress reduced the Pn of *Oryza sativa* under steady high light but had a weak impact on Pn under steady low light [34]. Interestingly, drought stress did not affect the time required to reach 50 or 90% of the maximum Pn after transitioning from low light to high light, but significantly decreased Pn in the low-light stage of changing light [34]. Sakoda et al. reported that drought stress delayed photosynthetic induction in *Oryza sativa* and *Glycine max* but only slightly interfered with maximum Pn values under steady high light [35]. Both studies emphasized the limitation of stomatal factors on dynamic photosynthesis under drought stress [34,35]. Sun et al. reported that drought stress delayed photosynthetic induction in *Solanum lycopersicum* [36].

Zhang et al. showed that salt stress did not affect Pn under steady high light but decelerated photosynthetic induction in *Solanum lycopersicum* [37]. However, two years later, the group reported an opposite result: salt stress suppressed Pn under steady high light but did not affect photosynthetic induction and Pn in *Solanum lycopersicum* [30]. This difference may have been caused by different salt concentrations and treatment durations.

In summary, dynamic photosynthesis is more sensitive to drought and salt stresses than steady photosynthesis; drought and salt stresses significantly increase the photosynthetic loss caused by changing light.

### 2.3. Air Temperature and Air Humidity

High temperature inhibited Pn more seriously under changing light than steady light conditions in *Shorea leprosula* and *Oryza sativa* seedlings [38,39]. High temperature also decelerated photosynthetic induction in *Oryza sativa* [40]. Research by Kang et al. showed that the effect of high temperature on photosynthetic induction was species-dependent: raising the temperature from 30 °C to 40 °C decelerated photosynthetic induction in *Croton argyratus, Shorea leprosula* and *Lepisanthes senegalensis*, but accelerated photosynthetic induction in *Neobalanocarpus heimii* [41].

Kaiser et al. reported that a decrease in temperature from 30.5 °C to 15.5 °C mildly delayed photosynthetic induction in *Solanum lycopersicum* [42]. The same study showed that raising leaf-to-air vapor pressure deficits from 0.5 to 2.3 kPa did not affect photosynthetic induction in *Solanum lycopersicum* [42]. In contrast, Tinoco-Ojanguren and Pearcy reported that a higher leaf–air vapor pressure deficit did not affect the photosynthetic carbon assimilation under steady high or low light, but significantly delayed photosynthetic induction in two rainforest *Piper* species [17].

Hence, dynamic photosynthesis is more sensitive to suboptimal temperature and humidity than steady photosynthesis, and the response of dynamic photosynthesis to suboptimal temperature and humidity is species dependent.

### 2.4. Carbon Dioxide Concentration

Elevated carbon dioxide concentrations led to an increase in the induction state 60 s after leaf illumination by 129% and 108% in broadleaved *Fagus sylvatica* and coniferous *Picea abies*, respectively, but elevated carbon dioxide concentrations decreased the time required to reach 90% of the maximum Pn by 5–15% and 21–28% in *Fagus sylvatica* and *Picea abies*, respectively, indicating that the acceleration during the initial stage of photosynthetic initiation was more significant than that during the later stage of photosynthetic initiation [43]. *Populus* species grown under conditions of higher carbon dioxide had a higher photosynthetic induction state, a shorter induction time, and reduced induction limitation to photosynthetic carbon assimilation after the plants were transferred from low to high light under growth carbon dioxide concentration [44]. A similar result was observed in *Shorea leprosula* [45]. Temporarily elevated carbon dioxide concentrations also accelerated photosynthetic induction and photosynthetic carbon assimilation under changing light conditions in *Dipterocarpus sublamellatus* [46] and *Solanum lycopersicum* [47]. Temporary low carbon dioxide (20 Pa) levels delayed photosynthetic induction in *Solanum lycopersicum* [42]. Kang et al. reported that both long-term and temporarily elevated carbon dioxide concentrations accelerated photosynthetic induction, though the acclimation of plants to high carbon dioxide reduced the acceleration of photosynthetic induction [48]. The above results clearly indicate that increased carbon dioxide concentrations not only increase the maximum photosynthetic capacity under steady high light, but also reduce the loss in Pn caused by changes in light intensity.

### 2.5. Nitrogen Nutrition

High nitrogen supply accelerated photosynthetic induction in Solanum lycopersicum and Zea mays [49,50]. A greater nitrogen supply improved the rapid response of Pn to changing light in Oryza sativa; the loss in Pn caused by changing light was relieved by increased nitrogen supply [39,51]. However, Li et al. observed the opposite result in Glycine max: although reducing nitrogen supply reduced photosynthesis under steady light, it alleviated the loss in Pn in changing light compared with that in steady light, which was attributed to the relative excess of RuBP regeneration-related enzymes in low-nitrogen leaves [52]. The effect of other nutrients on dynamic photosynthesis under changing light is still unclear, although many studies have shown that various nutrients significantly affect photosynthesis under steady light [53,54,55].

### 2.6. Circadian Rhythm

Dynamic photosynthesis under changing light is more sensitive to circadian rhythms than steady photosynthesis. In *Arabidopsis thaliana* planted in a changing light regime, the time constant for the light-saturated rate of carbon assimilation was highest in the evening and lower in the morning; the lowest value was recorded at midday [29]. Küppers et al. showed that the photosynthetic induction in *Pothos scandens* was faster in the early morning and at night than that at midday, and that the photosynthetic induction in another species, *Hydnocarpus pentandra*, was faster at night than in the morning and afternoon [18]. Poorter and Oberbauer reported that photosynthetic induction in *Dipteryx panamensis* and *Cecropia obtusifolia* with different successional traits was much faster in the morning than in the afternoon; however, the light-saturated steady-state photosynthetic rate was consistent at different times of day [31]. A similar result was reported by Robert and Pearcy [56,57]. These research works indicate that the effect of circadian rhythms on dynamic photosynthesis is species dependent.

## 3. Effects of Non-Environmental Factors on Dynamic Photosynthesis under Changing Light

### 3.1. C3 and C4 Photosynthetic Type

Pearcy et al. first studied dynamic photosynthesis under changing light in C3 and C4 tree species [58]. They observed that the effect of light change on Pn was similar in C3 and C4 tree species. Later, Pearcy and his colleagues analyzed the photosynthesis of the C4 species *Zea mays* under lightfleck [59] and compared it with selected C3 species [57,60,61]. It was observed that maize utilized lightfleck more efficiently than the C3 species. Kubásek et al. reported that the carbon assimilation loss and growth slowdown caused by changing light were more serious in two C4 species than in two C3 species [62]. The comparison of 18 C3 and C4 species, including C3, C4, C4-like and C3–C4 intermediate (C2) species in the *Flaveria* genus, showed that C4 plants have a significantly lower photosynthetic utilization efficiency of changing light than C3 plants; in addition, the C4-like *Flaveria* species had similar behavior to that of C4 *Flaveria* species, and the C3–C4 intermediate *Flaveria* species had similar behavior to that of the C3 *Flaveria* species [63]. However, Lee et al. reported the opposite result: six C4 bioenergy grasses utilized changing light better than six C3 bioenergy grasses [64]. Recent research in three phylogenetically linked pairs of C3 and C4 species from the *Alloteropsis*, *Flaveria* and *Cleome* genera indicated that photosynthetic induction was slower in C4 than in C3 species [65]. Zheng also reported that photosynthetic induction was much faster in the C4 species *Amaranthus tricolor* than in the C3 species *Glycine max*; in addition, this research reported that short-term elevated temperature and carbon dioxide conditions promoted photosynthetic induction in the C3 species *Glycine max*, but not in the C4 species *Amaranthus tricolor* [66].

**Figure 2:**
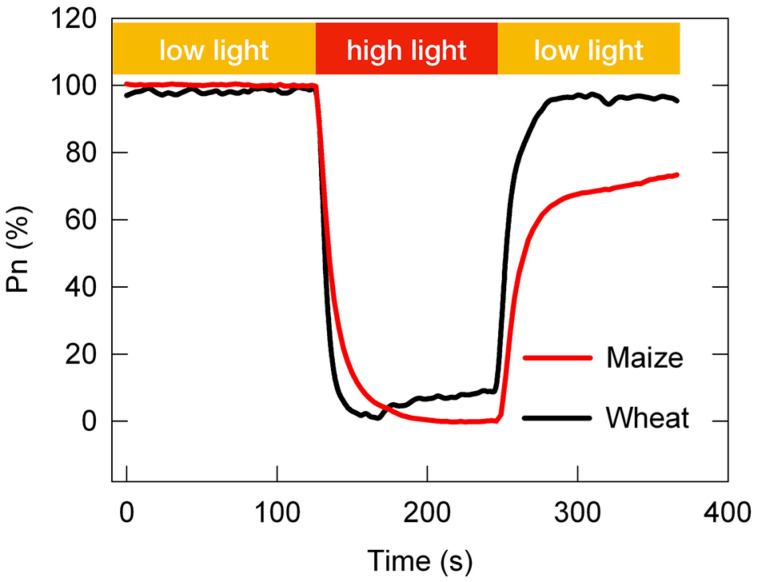
The dynamic net photosynthetic carbon assimilation rate (Pn) in leaves of the C3 species *Triticum aestivum* and the C4 species *Zea mays*. The relative Pn curve was obtained from the standardization between the Pn under constant high light (100%) and the minimum Pn under constant low light (0%).

At present, the adaptability of C3 and C4 plants to changing light is still an open question. As shown in Figure 2, the post-irradiance carbon burst was negligible in NADP-ME-type C4 species, and the post-irradiance carbon assimilation was higher in NADP-ME-type C4 species than in C3 species; however, the loss in photosynthetic carbon assimilation in the photosynthetic induction stage was much greater in NADP-ME-type C4 species than in C3 species [63,64]. In other words, compared with C3 plants, C4 plants fix more carbon dioxide during low-light periods of changing light, while C4 plants fix less carbon dioxide during high-light periods of changing light. Some scholars propose that C4 photosynthesis may be both more and less resilient than C3 to changing light conditions, depending on the frequency at which these light changes occur [8]. They speculate that C4 photosynthesis may have more advantages than C3 photosynthesis under high-frequency changing light (10–15 s or less).

Most studies on C4 plants have focused on their advantages over C3 plants, pointing to their high-temperature resistance, water conservation ability and high yield, and have proposed grand plans for engineering C4 photosynthesis into C3 plants [67,68], including the “C4 rice project” [69]. We believe it is necessary to pay attention to the photosynthetic light utilization efficiency of C4 plants under changing light conditions, as this will determine whether C4 photosynthesis is more advantageous than C3 photosynthesis in high-density crop canopies with intense changes in light intensity.

### 3.2. Inter-Specific Variations

Valladares et al. reported that “leaves of understory species showed the most rapid induction and remained induced longer once transferred to the shade than did leaves of medium- or high-light species” [70]. Leakey et al. reported that photosynthetic induction was faster in shade-tolerant dipterocarp species than in less shade-tolerant species [24]. Early successional species showed the slowest photosynthetic induction, while mid-successional species showed the fastest photosynthetic induction, and the rate of photosynthetic induction in late-successional species was between those of early-successional and mid-successional species [71]. Küppers et al. showed that late-successional species had faster photosynthetic induction in continuous saturated light following darkness, and a slower loss in induction in darkness following high light than early-successional species [18].

It was reported that photosynthetic induction was faster in four fern species than in three woody species [33]. The loss in photosynthetic carbon assimilation during photosynthetic induction was less in fern species than in gymnosperm and angiosperm species [72].

In general, shade-tolerant species and late-successional species have a higher photosynthetic energy utilization efficiency under changing light than heliophilic species and early successional species.

### 3.3. Intra-Specific Variations

In order to improve the photosynthetic light energy utilization efficiency of crops by utilizing natural genetic variation, dynamic photosynthesis under changing light was compared among various cultivars of crop species, including *Oryza sativa* [73,74,75,76], *Oryza glaberrima* [77], *Triticum aestivum* [78], *Glycine max* [79,80], *Musa nana* [81], *Manihot esculenta* [82], *Brassica napus* [83] and *Rosa rugosa* [84].

Generally, the variation in photosynthetic carbon assimilation among cultivars in changing light conditions greatly exceeded that in steady light conditions [74,76,77,78,79,82,83,84]. Acevedo-Siaca et al. reported that although wild progenitors of *Oryza sativa* had lower Pn under steady high light than cultivated *Oryza sativa*, wild *Oryza sativa* assimilated 16– 40% more carbon dioxide than cultivated *Oryza sativa* [75]. Moreover, there was no significant correlation between steady and dynamic photosynthetic traits [76,80].

Researchers have also attempted to explain intra-specific variations in photosynthetic induction. Intra-specific variations in photosynthetic induction were mainly attributed to the activation rate of Rubisco [74,76,79,80]. Liu et al. reported that the rate of photosynthetic induction was positively correlated with the nitrogen content and Rubisco content in leaves of *Brassica napus* [83]. Soleh et al. also reported the activation rate of Rubisco was related to the single-nucleotide polymorphisms of the Rubisco activase gene [79]. In contrast, research on *Oryza sativa* [85], *Musa nana* [81] and *Manihot esculenta* [82] showed that stomatal conductance was the major limitation to dynamic photosynthesis under changing light conditions. Adachi et al. reported that the faster induction response to sudden increases in light intensity in a high-yield *Oryza sativa* cultivar (Takanari), compared with a cultivar with lower yield (Koshihikari), was explained in part by the maintenance of a larger pool of Calvin–Benson cycle metabolites in Takanari [73].

### 3.4. Stomatal Behavior

The response rate of stomatal opening to changes in light intensity is slower than that of photosynthetic carbon assimilation [7,86]. Pre-opening stomata under low light or darkness could accelerate photosynthetic induction under high-light conditions [32,56,57,87,88]. However, recent research showed that pre-dawn stomatal opening does not substantially enhance photosynthetic induction in *Helianthus annuus* [89]. It has also been reported that smaller and denser stomata contributed to a faster stomatal response under changing light, resulting in improved photosynthetic induction [85,90,91]. The stomatal closure rate also affects dynamic photosynthesis under changing light conditions. Delayed stomatal closure, similar to stomatal pre-opening, accelerates photosynthetic induction following an increase in light intensity. However, it also increases water loss, and limits long-term photosynthetic carbon assimilation under water-restricted conditions [92].

More direct evidence on the contribution of stomata to photosynthetic carbon assimilation under changing light comes from comparisons of dynamic photosynthesis between wild-type and transgenic or mutant plants with specifically modified stomatal characteristics. The abscisic acid-deficient *flacca* mutant of *Solanum lycopersicum* displayed very high stomatal conductance and faster photosynthetic induction than the wild-type [93]. The STOMAGEN-overexpressing *Arabidopsis thaliana* line (ST-OX) and the *EPIDERMAL PATTERNING FACTOR 1* knockout line (*epf1*) showed faster photosynthesis induction than the wild-type after a step increase in light, owing to their higher stomatal conductance under initial dark conditions; however, there were no significant variations in photosynthetic carbon assimilation under steady light between the wild-type and ST-OX or *epf1* [94]. *SLAC1* encodes a stomatal anion channel that regulates stomatal closure; a deficiency of *SLAC1* in *Oryza sativa* and *Arabidopsis thaliana* significantly accelerated stomatal opening, photosynthetic induction and plant growth in changing light conditions [95,96]. *Open stomata 1* (*ost1*) mutants always open stomata even under darkness; the *PROTON ATPASE TRANSLOCATION CONTROL 1* overexpression line (*PATROL1*) closed and opened stomata faster than wild-type *Arabidopsis thaliana*; hence, *ost1* and *PATROL1* assimilated more carbon dioxide and accumulated more dry weight under changing light conditions rather than in constant light [96]. The *BLINK1* gene encodes a light-gated K^+^ channel in guard cells. *BLINK1* overexpression in *Arabidopsis thaliana* accelerated both stomatal opening under light exposure and closing after irradiation; moreover, it drove a 2.2-fold increase in biomass in changing light conditions [97]. 

The above results unanimously indicate that reducing stomatal limitations can effectively improve photosynthetic carbon assimilation, especially under changing light conditions, although it may lead to a decrease in water-use efficiency. Therefore, the most effective method is to accelerate the response rate of stomata to changes in light intensity, rather than increasing the opening of stomata.

### 3.5. Other Genes

In addition to the genes mentioned above that dominate stomatal development and affect dynamic photosynthesis under a changing light environment, researchers have also found more genes that influence dynamic photosynthesis.

Non-photochemical quenching (NPQ) is an important photoprotection mechanism of PSII, and even of PSI; it gradually activates under light and relaxes in darkness and low light [98]. Modeling analysis predicted that accelerating the relaxation of NPQ during transferal from high to low light could enhance photosynthetic carbon assimilation under low-light conditions [99,100]. Compared with the wild-type, NPQ was enhanced and the photosynthetic induction was decelerated with PsbS gene overexpression in *Oryza sativa*, a gene which encodes a key protein of NPQ; the opposite result occurred in RNAi lines [101]. Surprisingly, the simultaneous overexpression of three key genes of NPQ, PsbS, violaxanthin deepoxidase (VDE) and zeaxanthin epoxidase (ZE), accelerated the relaxation of NPQ and enhanced post-irradiance carbon assimilation, thus improving biomass and yield in changing light conditions. Similar phenomena were observed in *Nicotiana tabacum* [102] and *Glycine max* [103]. Unfortunately, similar genetic improvements failed to improve, and even suppressed photosynthetic carbon assimilation and dry matter accumulation in *Arabidopsis thaliana* [104] and *Solanum tuberosum* [105]. These results indicate that the effects of accelerated NPQ relaxation on photosynthesis and growth are species-dependent.

Overexpression of the flavodiiron gene from *Physcomitrium patens* in *Arabidopsis thaliana* enhanced growth in changing light conditions by increasing photosynthetic carbon assimilation in the high-light phase of changing light conditions [106]. The deregulation K^+^ exchange antiporter 3 (KEA3) gene in *Arabidopsis thaliana* increased photosynthetic carbon assimilation during photosynthetic induction [107].

Overexpression of the Rubisco activase (RCA) gene accelerated photosynthetic induction in *Oryza sativa*, especially at high temperatures [40]. Researchers modified the gene sequence of RCA at different sites to render RCA continuously activated, even in the dark; the transformant of *Arabidopsis thaliana* that expresses modified RCA had accelerated photosynthetic induction and enhanced growth in changing light [108,109]. Moreover, McCormick and Kruger reported that the mutant of the cytosolic 6-phosphofructo-2-kinase/fructose-2,6-bisphosphatase (F2KP) gene had normal Pn under steady light but exhibited delayed photosynthetic induction [110]. The C4 species *Setaria viridis* overexpressed pyruvate phosphate dikinase regulatory proteins (PDRP) and kept pyruvate phosphate dikinase (PPDK) active even under darkness but delayed photosynthetic induction, which is due to the exhaustion of pyruvate [111].

Recent research has shown that the Pn of a chlorophyll-deficient *Glycine max* mutant was similar under steady light but was more responsive to changing light than wild-type *Glycine max* [112]. However, Ferroni et al. reported that chlorophyll-depleted mutants and wild-type *Triticum aestivum* had comparable adaptation to changing light [113].

Researchers have improved photosynthetic carbon assimilation under changing light conditions through a variety of synthetic biology methods. However, further research is needed to determine whether these methods can be extended to different crops.

## 4. Conclusions and Challenges of Photosynthetic Research under Changing Light

In recent years, more and more attention has been paid to dynamic photosynthesis under changing light, and the study of dynamic photosynthesis has expanded from understory plants to crops. Researchers have studied dynamic photosynthesis from more perspectives such as ecology, agriculture, plant physiology, molecular biology, synthetic biology, etc. It has been shown that dynamic photosynthesis is more sensitive to environmental and non-environmental factors than steady photosynthesis. Hence, in order to cope with more adverse environments in the future, further research on dynamic photosynthesis should be performed. Improving the efficiency of photosynthetic light utilization is considered an effective way to increase crop yield. However, the homogenization of photosynthetic capacity (under steady high light) of major crops limited the breeding of high-yield varieties [114,115]. Photosynthetic carbon assimilation under changing light is more diverse between species and cultivars than that under steady high light; hence, it is promising to breed cultivars with higher photosynthetic light utilization under changing light conditions.

Dynamic photosynthesis during the high-light stage of a changing light regime was determined by both stomatal and non-stomatal factors, while the main factor affecting photosynthesis during the low-light stage of a changing light regime was the light reaction. Although knowledge of the limiting factors of dynamic photosynthesis has greatly increased and dynamic photosynthesis has been successfully improved by synthetic biology, these mechanisms and strategies should be verified in more species and various environments, as they may be species- and environment-dependent.

We also propose four challenges for research in dynamic photosynthesis under changing light:

(1) Low efficiency of gas exchange measurement technology. Photosynthetic gas exchange technology is the “gold standard” for studying dynamic photosynthetic carbon assimilation, but it is very time-consuming. For high-throughput measurements, multiple instruments need to be used in one experiment, but such instruments are expensive. Although chlorophyll fluorescence imaging technology has been used in high-throughput analysis of photosynthetic performance, it has reduced measurement accuracy. In addition, not all electrons are consumed for photosynthetic carbon assimilation, so it is uncertain whether chlorophyll fluorescence is able to completely reflect the difference in photosynthetic carbon assimilation.
(2) The measurement procedures of dynamic photosynthesis are not uniform. Although we believe that the measurement procedure selected for each experiment is reasonable and most suitable for experimentation, different measurement procedures limit the comparison between various experiments. For example, some studies measured photosynthetic induction after darkness- or low-light-adapted leaves were exposed to high light [96], while other studies measured Pn after high-light-adapted leaves were transferred to fluctuating light with light intensities alternating between low and high light [52,63], and yet other studies measured Pn under natural light fluctuations [95]. At present, there are uniform measurement procedures in research concerning steady photosynthesis, including light intensity and intercellular carbon dioxide concentration response curves of the net photosynthetic rate. We propose that colleagues discuss and design simple and representative measurement procedures for dynamic photosynthesis and include these in their own research.
(3) What parameter should be used to express the rate of photosynthetic induction? There are two options: one is the absolute value of Pn after high light appears, namely the absolute photosynthetic induction rate, and the other is the time required for Pn to reach 90% or 50% of its maximum value under steady high light, namely, the relative photosynthetic induction rate. Sometimes, both parameters reach the same conclusion; for example, in one study, with increasing carbon dioxide concentration, the absolute value of Pn during photosynthetic induction increased, and the time required for Pn to reach its maximum decreased [47]. However, in other research, different parameters may lead to opposite conclusions. For example, Durand et al. reported that the Pn of shaded leaves was constantly lower than that of sunned leaves during photosynthetic induction; however, the time required for Pn to reach its maximum was shorter in shaded leaves than in sunned leaves [21]. Most research has favored the relative photosynthetic induction rate, which often leads to paradoxical results wherein a lower maximum Pn is usually accompanied by a shorter time required to reach maximum Pn, and such plants are judged to be “able to efficiently utilize changing light”. Therefore, we suggest avoiding using only relative photosynthetic induction rate in research and, instead, opting to use both absolute and relative photosynthetic induction rates.
(4) Varying light intensity changes in relation to environmental factors, such as temperature and relative humidity. Previous research generally only investigated changes in the light intensity while keeping other environmental factors constant. Of course, such experimental designs conformed to the “single factor variable” so as to focus on the impact of changing light intensity on photosynthesis. However, this is only a poor approximation of natural phenomena. Recently, Kang et al. made a useful attempt to address this. Their research shows that “concurrent increases in leaf temperature with light accelerate photosynthetic induction in tropical tree seedlings” [39].

## Data Availability

The data presented in this study are available on request from the corresponding author.
